# Early recognition and risk stratification of cardiac rupture after acute myocardial infarction: from clinical red flags to emerging biomarkers

**DOI:** 10.3389/fcvm.2026.1813572

**Published:** 2026-09-18

**Authors:** Dan Zhang, Jing Chen, Xiaosu Wang, Jingdan Yu, Bo Liu

**Affiliations:** 1 Department of Clinical Laboratory, Wuhan Asia General Hospital, Wuhan Asia General Hospital Affiliated to Wuhan University of Science and Technology, Wuhan, China; 2 Department of Critical Care Medicine, Wuhan Asia General Hospital, Wuhan Asia General Hospital Affiliated to Wuhan University of Science and Technology, Wuhan, China

**Keywords:** acute myocardial infarction, artificial intelligence, biomarkers, cardiac rupture, echocardiography, intramyocardial hemorrhage, mechanical complications, risk stratification

## Abstract

Cardiac rupture (CR) remains among the most lethal mechanical complications of acute myocardial infarction (AMI), with in-hospital mortality exceeding 50%. Although primary percutaneous coronary intervention has reduced CR incidence to 1%–2%, mortality has not declined proportionally because the abrupt onset of rupture limits the opportunity for intervention. The early post-infarction period—particularly the first 72 h for free wall rupture and extending through day 7 for ventricular septal and papillary muscle rupture—represents the critical window for detection. This mini review synthesizes clinical warning signs, point-of-care imaging findings, and circulating biomarker trajectories that may herald impending CR, organized by temporal window and rupture subtype. We evaluate emerging biomarkers reflecting inflammatory, extracellular-matrix-degradation, and myocardial-stress pathways, as well as artificial intelligence–assisted prediction tools. Building upon the prediction model synthesis by Mao et al. within this Research Topic, we propose a four-step risk stratification algorithm integrating baseline clinical predictors, dynamic three-track surveillance (clinical, imaging, and biomarker), red-flag–triggered risk re-classification, and subtype-specific diagnostic pathways. This framework is designed to bridge the translational gap between statistical risk estimation and actionable bedside recognition during the critical post-AMI period.

## 1 Introduction

Cardiac rupture comprises three subtypes with distinct pathological mechanisms and temporal profiles: free wall rupture (FWR, ∼70% of cases), ventricular septal rupture (VSR, ∼20%), and papillary muscle rupture (PMR, ∼10%) (1, 2). FWR exhibits a bimodal peak within the first 24 h and at 3–5 days, VSR predominantly occurs at 3–7 days, and PMR peaks between 2 and 7 days post-AMI (3). These differences mandate a subtype-aware approach to early recognition. The clinical characteristics and outcomes of CR have also been examined in large registries and longitudinal hospital cohorts (31–33).

In a systematic review and meta-analysis published within this Research Topic, Mao et al. (4) synthesized 10 prediction models with a pooled concordance statistic of 0.83, identifying age, sex, Killip class, left ventricular ejection fraction, and PCI status as robust predictors. Earlier studies by Qian et al. (5) and Fu et al. (6) similarly constructed clinically applicable risk scores. However, these models fundamentally address *who* is at risk, rather than *what* specific clinical signs, imaging findings, and biomarker trajectories clinicians should monitor once a high-risk patient is identified. This translational gap—from statistical risk estimation to actionable bedside recognition—constitutes a critical unmet need.

This mini review aims to bridge this gap by: (1) synthesizing clinical, imaging, and biomarker warning signs of impending CR organized by temporal window and rupture subtype; (2) evaluating emerging biomarkers and artificial intelligence–assisted prediction tools; and (3) proposing a multi-dimensional risk stratification algorithm for bedside application. By addressing the translational step from risk identification to bedside recognition, this review complements the prediction model synthesis by Mao et al. (4) and provides a clinician-oriented framework for early CR detection in the reperfusion era.

## 2 Clinical red flags and bedside recognition

### 2.1 Hemodynamic and clinical warning signs

The clinical presentation of cardiac rupture varies dramatically depending on the subtype and acuity of the event. Acute free wall rupture (FWR) typically manifests as sudden hemodynamic collapse with electromechanical dissociation (EMD), often within the first 24 h post-AMI (2, 7). The classic Beck's triad—hypotension, jugular venous distension, and muffled heart sounds—may be present but is frequently absent in precipitous cardiovascular collapse. In-hospital mortality exceeds 80%, with diagnosis often confirmed only at autopsy or during emergent surgery (7). Any unexplained sudden hemodynamic deterioration should trigger immediate bedside echocardiography.

In contrast, subacute or “oozing” FWR (Becker type II–III) offers a critical diagnostic window. These patients characteristically present with recurrent chest pain of pleuritic quality—distinct from the initial ischemic pain—accompanied by transient hypotensive episodes with spontaneous recovery, a pattern described as “stuttering” hemodynamic instability (2, 3). Nausea, restlessness, and new ST-segment re-elevation may herald an evolving contained rupture. Crucially, the subacute presentation represents the window in which timely recognition can alter outcomes, as contained rupture may allow sufficient time for surgical repair.

Ventricular septal rupture (VSR) and papillary muscle rupture (PMR) present with distinct hemodynamic signatures. VSR is characterized by the abrupt onset of a harsh holosystolic murmur at the left sternal border, often accompanied by a palpable precordial thrill. Hemodynamic confirmation relies on demonstrating an oxygen saturation step-up from the right atrium to the right ventricle; data from the UK national registry confirmed that early recognition and hemodynamic stabilization remain the key determinants of survival (2, 8). PMR typically presents with a new systolic murmur at the apex—which may be deceptively soft or absent in severe acute mitral regurgitation—together with flash pulmonary edema and prominent V waves on pulmonary capillary wedge pressure monitoring (2, 9). The temporal profiles described in the Introduction should guide time-dependent clinical vigilance for the most probable rupture subtype at each stage of the post-infarction course.

### 2.2 Point-of-care imaging for early detection

Bedside echocardiography is the cornerstone modality for early detection of cardiac rupture and should be regarded as a first-line diagnostic tool in any patient with suspected mechanical complications (2). New or increasing pericardial effusion is the single most important imaging predictor of impending FWR and has been incorporated into validated prediction models (5). Quantification is essential: a circumferential effusion that is new, rapidly progressive, or moderate or greater in size warrants immediate escalation, although no validated absolute threshold has been established specifically for CR prediction. For VSR, color Doppler imaging enables direct visualization of trans-septal flow and shunt estimation, while in PMR, echocardiography permits assessment of mitral regurgitation severity and flail leaflet morphology (2, 7). Progressive wall motion abnormalities extending beyond the initial infarct territory may serve as an additional warning sign.

In hemodynamically stable patients with equivocal echocardiographic findings, cardiac computed tomography angiography can delineate contained rupture or pseudoaneurysm through identification of contrast extravasation, intramyocardial dissection tracks, or hematoma formation (2, 10).

Cardiac magnetic resonance (CMR) imaging has emerged as a powerful tool for identifying tissue characteristics associated with rupture vulnerability. Lechner et al. demonstrated that the coexistence of microvascular obstruction (MVO) and intramyocardial hemorrhage (IMH) defines a high-risk phenotype associated with significantly higher major adverse cardiovascular events (MACE) rates (19.5% vs. 4.4%; *P* < 0.001), although this endpoint encompassed adverse outcomes beyond CR specifically (11). Whether this MVO + IMH phenotype specifically predicts CR—as opposed to the broader spectrum of adverse post-STEMI remodeling—has not been directly tested and represents an important area for future investigation. Mechanistically, IMH extends as a “wave front” of reperfusion injury that compromises myocardial salvage, providing a pathophysiological link between microvascular damage and structural vulnerability (12, 13). Although CMR is not feasible in acutely unstable patients, it holds promise for risk stratification in stabilized post-reperfusion patients. Based on the known temporal distribution of CR subtypes and clinical pragmatism, we suggest a serial echocardiographic surveillance protocol for high-risk patients: focused bedside echocardiography at 6, 24, 48, and 72 h post-AMI, with specific attention to pericardial effusion, septal integrity, and mitral valve competence. These intervals are aligned with the bimodal FWR peak distribution and standard coronary care unit shift schedules, although the optimal surveillance frequency has not been empirically established and warrants prospective evaluation.

### 2.3 Biomarker profiles for risk stratification

Circulating biomarkers provide an additional dimension for cardiac rupture risk stratification. Elevated white blood cell count and neutrophil-to-lymphocyte ratio (NLR) reflect the intensity of the post-infarction inflammatory response that drives matrix metalloproteinase (MMP) activation and extracellular matrix (ECM) degradation (3). C-reactive protein (CRP) trajectory is particularly informative: persistent elevation beyond 72 h, rather than the expected post-infarction decline, suggests ongoing inflammatory destruction and may herald impending mechanical failure. In their meta-analysis, Mao et al. reported a positive but non-significant pooled association between white blood cell count and CR risk (OR 1.47, 95% CI 0.46–4.71, I² = 95%), with the substantial heterogeneity likely attributable to differences in sampling time points across studies (4). Of note, Tiller et al. recently demonstrated that inflammatory biomarker profiles correlate with IMH severity post-STEMI, further supporting the link between persistent inflammation and myocardial structural vulnerability (14).

Among markers of ECM degradation, matrix metalloproteinase-9 (MMP-9) is the most mechanistically relevant to CR and peaks at 24–48 h post-AMI—coinciding with the initial rupture vulnerability window; its role in the CARD9–LCN2–MMP-9 cascade is discussed in Section 3.1 (2, 3). B-type natriuretic peptide (BNP) and N-terminal pro-BNP serve as surrogates for ventricular wall stress, with markedly elevated levels correlating with larger infarct size and heightened rupture risk in observational studies—although, as with most biomarkers discussed herein, this association derives primarily from general post-AMI cohorts rather than CR-specific outcome data. A secondary troponin rise after initial decline may indicate infarct extension or evolving mechanical failure. Longitudinal CMR data confirm that tissue injury markers at the acute phase predict progressive adverse remodeling—though this evidence pertains to broader post-STEMI outcomes rather than CR specifically—underscoring the prognostic value of early biomarker assessment (15). Importantly, no single biomarker currently achieves sufficient diagnostic accuracy for CR prediction in isolation, motivating the exploration of novel candidates discussed in the following section. The principal clinical, echocardiographic, biomarker, and electrocardiographic warning signs are summarized by time window and rupture subtype in Table 1.

**Table 1:** Warning signs of cardiac rupture organized by time window and rupture subtype. Synthesized from references (2, 3, 7).

Domain	Hyperacute (<24 h)	Acute (1–7 days)	Subacute (>7 days)
Clinical signs	Sudden hemodynamic collapse; EMD; Beck's triad; cardiogenic shock	Recurrent pleuritic chest pain; new murmur; transient hypotension (“stuttering” pattern); restlessness	Progressive heart failure; persistent hypotension; new murmur with delayed onset
Echo findings	Acute pericardial effusion; new severe regional WMA; RV compression	Increasing pericardial effusion; new VSD flow on color Doppler; worsening MR; pseudoaneurysm	Contained rupture; organized effusion; flail mitral leaflet; chronic VSD with Qp/Qs elevation
Biomarker pattern	WBC spike; markedly elevated NLR; CRP surge; early MMP-9 peak	Persistent CRP elevation (>72 h); secondary troponin rise; declining albumin; BNP surge	Chronic inflammatory markers; sustained BNP elevation; fibrosis markers (galectin-3, sST2)
ECG changes	ST re-elevation; abrupt bradycardia; new conduction block	Persistent ST elevation; new T-wave inversion; transient ST re-elevation	Non-specific ST-T changes; persistent Q waves with new low voltage
Predominant subtype	FWR (acute; Becker type I)	VSR > subacute FWR (Becker type II–III)	PMR > contained FWR

EMD, electromechanical dissociation; WMA, wall motion abnormality; RV, right ventricle; VSD, ventricular septal defect; MR, mitral regurgitation; Qp/Qs, pulmonary-to-systemic blood flow ratio; WBC, white blood cell count; NLR, neutrophil-to-lymphocyte ratio; CRP, C-reactive protein; MMP-9, matrix metalloproteinase-9; BNP, B-type natriuretic peptide; sST2, soluble suppression of tumorigenicity 2; FWR, free wall rupture; VSR, ventricular septal rupture; PMR, papillary muscle rupture.

## 3 Emerging biomarkers and novel stratification tools

### 3.1 Novel circulating biomarkers

Given the extremely low incidence of CR (<2% of AMI patients), no prospective study has evaluated circulating biomarkers with CR itself as a pre-specified primary endpoint. The biomarkers discussed below have been validated primarily for broader post-AMI adverse outcomes (MACE, mortality, heart failure); their relevance to CR is inferred on the basis of biological plausibility—that is, their mechanistic involvement in the pathways known to underlie myocardial structural failure. This indirect evidence strategy is acknowledged as a limitation, and dedicated prospective validation for CR prediction is urgently needed.

Galectin-3 (Gal-3), a β-galactoside–binding lectin secreted by activated macrophages, independently predicted death and heart failure—though not CR specifically—with incremental value beyond troponin and sST2 in a population-based study of 1,342 MI patients (16). Its mechanistic involvement in inflammatory and profibrotic signaling within the infarct border zone (17) provides biological plausibility for a potential link to CR that warrants dedicated investigation.

Growth differentiation factor-15 (GDF-15), a stress-responsive TGF-β superfamily member upregulated in ischemic cardiomyocytes, independently predicts mortality post-AMI but has not been evaluated for CR specifically (18). Soluble suppression of tumorigenicity 2 (sST2), the decoy receptor for interleukin-33, robustly predicts all-cause mortality and MACE in AMI patients (meta-analysis of 14 studies) (19); notably, Xu et al. within this Research Topic demonstrated that dynamic serial sST2 measurements more accurately predict MACE than single baseline values, underscoring the importance of temporal trajectory monitoring aligned with our proposed surveillance framework (20).

The MMP-9/TIMP-1 axis remains the most mechanistically relevant biomarker pathway for CR. MMP-9 directly degrades type I and III collagen within the infarct zone, and its activity peaks at 24–48 h post-AMI—precisely coinciding with the initial rupture vulnerability window (3). An imbalance favoring MMP-9 over its endogenous inhibitor TIMP-1 reflects unopposed ECM destruction. Upstream, the CARD9–LCN2–MMP-9 cascade has been identified as a regulatory pathway: in murine models, CARD9 deficiency attenuates post-infarction cardiac injury through reduced LCN2-mediated MMP-9 activation, positioning this axis as a mechanistically informative target (21). Within this Research Topic, Liu et al. demonstrated that heparanase (HPSE)—an enzyme that degrades heparan sulfate proteoglycans and activates latent MMPs—independently predicts MACE in STEMI patients post-PCI, adding another dimension to the ECM degradation biomarker portfolio (22).

Given that no single biomarker achieves sufficient sensitivity or specificity for CR prediction in isolation (4), we propose a hypothesis-generating multi-biomarker panel framework integrating three complementary pathophysiological axes: (i) an inflammator*y* axis (NLR combined with CRP trajectory), (ii) an ECM degradation axis (MMP-9 or HPSE), and (iii) a myocardial stress axis (sST2 or Gal-3 combined with BNP). The individual biomarkers in this panel have been validated for MACE prediction rather than CR specifically; their inclusion is predicated on biological plausibility rather than direct CR outcome data. Multi-biomarker strategies have demonstrated superior discrimination over single markers in post-AMI risk stratification for other adverse outcomes, providing a rationale for future investigation specifically targeting CR as the endpoint.

### 3.2 AI-assisted prediction and real-time monitoring

Artificial intelligence (AI) and machine learning (ML) algorithms offer an emerging paradigm for integrating the multidimensional data streams—clinical, imaging, and biochemical—that characterize the post-AMI setting. ML-based approaches have already demonstrated potential in detecting occlusion MI patterns with sensitivity surpassing expert interpretation (23) and predicting post-AMI mortality more accurately than traditional risk scores (24). Extending these concepts, AI-driven automated echocardiographic analysis could enable continuous quantification of pericardial effusion progression, wall motion deterioration, and valvular competence—key parameters in our proposed serial echo protocol (25, 26). Within this Research Topic, Yue et al. identified mitochondrial dynamics–related genes as potential AMI diagnostic biomarkers using ML approaches, supporting transcriptomics-based risk profiling (27). Importantly, none of these AI/ML tools have been validated specifically for CR prediction, and their application to this endpoint remains entirely exploratory.

Despite this promise, substantial barriers impede clinical translation. The extremely low event rate of CR (∼1%–2%) limits training data for ML algorithms. Standardized CR phenotyping across multicenter registries is lacking—a limitation also highlighted by the substantial heterogeneity in prediction model meta-analyses (4). Nearly all existing models derive from single-center retrospective cohorts and lack prospective external validation. Until AI-assisted tools achieve the robustness required for clinical deployment, clinician-driven structured risk assessment remains essential, motivating the integrated framework proposed below.

## 4 Toward an integrated risk stratification framework

As outlined in the preceding sections, individual clinical signs, imaging findings, and circulating biomarkers each possess limited sensitivity and specificity for cardiac rupture (CR) prediction when considered in isolation. The meta-analysis by Mao et al. confirmed that existing prediction models, while identifying relevant risk factors, have not been prospectively validated and lack integration with dynamic monitoring data (4). To bridge this gap, we propose a four-step multi-dimensional algorithm applicable from admission through the high-risk post-infarction period (Figure 1).

**Step 1—Baseline Risk Assessment (at admission).** Clinicians should systematically evaluate established CR predictors from meta-analytic data (4–6): age ≥70 years, female sex, Killip class III–IV, heart rate >100 bpm, left ventricular ejection fraction (LVEF) < 40%, anterior ST-elevation myocardial infarction (STEMI), and absence of emergency PCI. Timely PCI remains the strongest protective factor, with a pooled odds ratio of 0.37 (95% CI 0.20–0.70) in the Mao et al. meta-analysis (4). Pharmacological context—including early beta-blocker and RAAS inhibitor use (protective) versus corticosteroid or NSAID exposure (potentially risk-aggravating)—should be noted as baseline modifiers, although therapeutic optimization is beyond the scope of this review. As a pragmatic bedside triage tool, we illustrate a simplified, hypothesis-generating stratification: ≥3 risk factors defines high baseline risk, 1–2 defines intermediate risk, and 0 defines low risk; corresponding care pathways range from coronary care unit admission with continuous monitoring (high) to enhanced surveillance (intermediate) to standard post-AMI care (low). We acknowledge that this equal-weight approach is a simplification, as individual predictors carry substantially different effect sizes (e.g., emergency PCI: pooled OR 0.37 [95% CI 0.20–0.70] vs. female sex: pooled OR 2.43 [95% CI 1.92–3.07]); a weighted system based on pooled effect sizes represents a future priority.
**Step 2—Dynamic Monitoring (0–72 h; extended to day 7 for VSR/PMR).** Continuous risk assessment requires three parallel surveillance tracks. The initial 72 h represent the highest-risk period for FWR; however, because VSR predominantly occurs at days 3–7 and PMR peaks at days 2–7, surveillance should be maintained through day 7 in patients with risk factors for these subtypes. The *clinical track* entails systematic hemodynamic assessment every 4–6 h, with particular attention to pain recurrence, new murmurs, and signs of tamponade or acute heart failure. The *imaging track* implements the serial echocardiographic protocol proposed in Section 2.2, with additional imaging at days 5 and 7 for patients at intermediate or high risk for VSR/PMR; microvascular injury patterns identified by CMR (when feasible) further refine risk stratification (11). The *biomarker track* comprises daily white blood cell count and NLR, C-reactive protein every 24 h, cardiac troponin trajectory, and BNP; where available, serial sST2 measurements provide additional prognostic information, as demonstrated by Xu et al. within this Research Topic (20). It should be noted that the thresholds and trajectories for these biomarkers have been established for broader post-AMI adverse outcomes; CR-specific cut-off values remain to be defined.
**Step 3—Integrated Risk Re-Classification.** The core principle is that CR risk is dynamic: baseline models estimate initial probability, but evolving data may fundamentally alter the risk trajectory. Red-flag triggers warranting immediate re-classification include: new or increasing pericardial effusion, secondary troponin rise after initial decline, persistent CRP elevation beyond 72 h, new systolic murmur, and unexplained hemodynamic instability. Patients initially classified as low or intermediate risk who develop any red flag should be upgraded to high risk with intensified surveillance. Those with high baseline risk who manifest any red flag enter a critical-risk category, triggering immediate multidisciplinary team activation including cardiac surgery consultation.
**Step 4—Subtype-Specific Diagnostic Pathways.** Once a mechanical complication is suspected, evaluation should follow subtype-specific pathways (2). For suspected FWR, urgent echocardiography targeting pericardial effusion is performed, with pericardiocentesis readiness and emergent cardiac surgery consultation. For suspected VSR, color Doppler imaging with oxygen saturation step-up guides shunt quantification (Qp/Qs), followed by consideration of mechanical circulatory support and repair—timing of which remains debated, as highlighted by the UK national registry experience (8). For suspected PMR, echocardiographic mitral regurgitation quantification and hemodynamic monitoring guide the urgency of surgical evaluation. This algorithm provides a structured, evidence-informed framework; prospective multicenter validation is required before clinical implementation.

**Figure 1:**
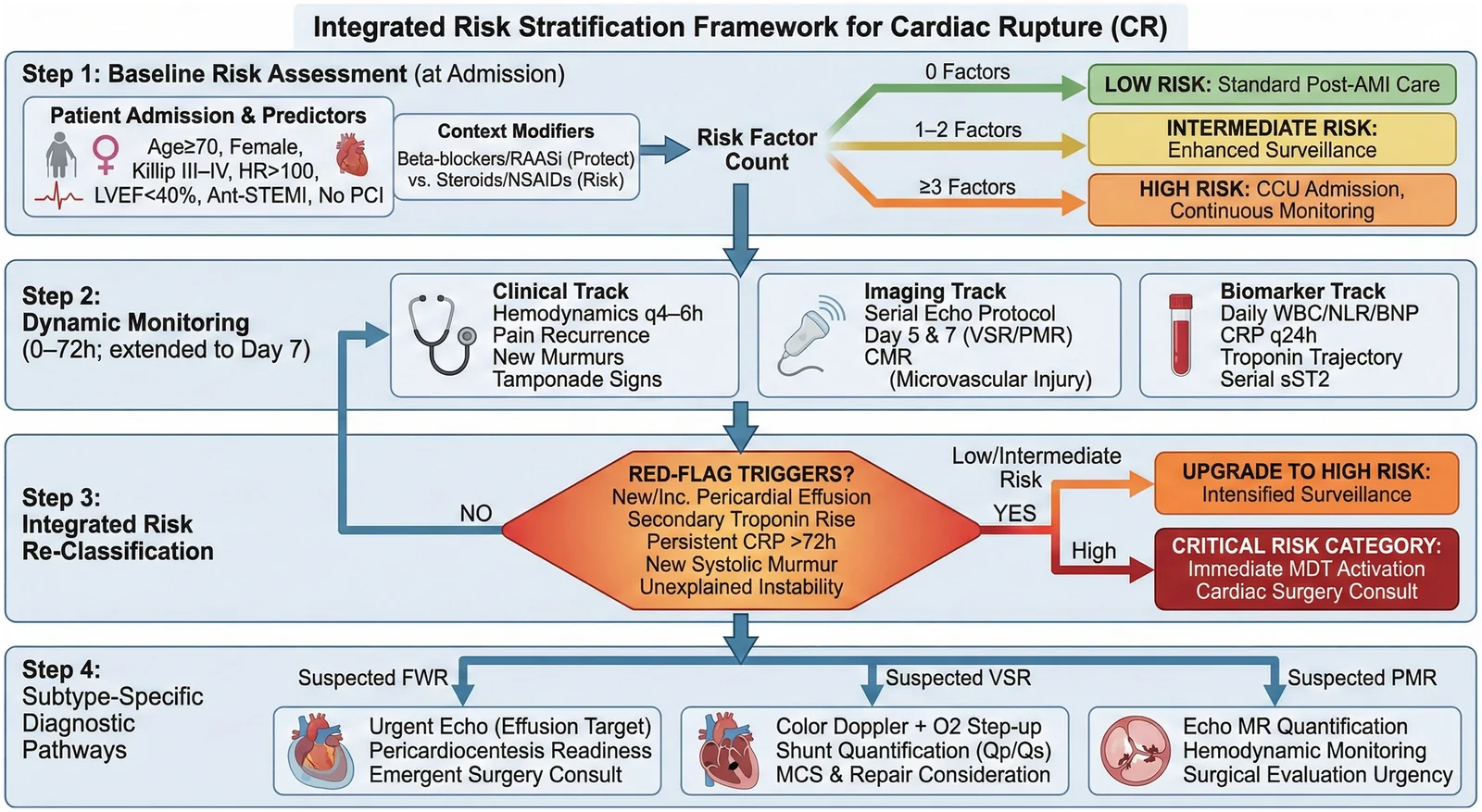
Multi-dimensional risk stratification algorithm for cardiac rupture after acute myocardial infarction. The proposed algorithm integrates four sequential steps: (Step 1) baseline risk assessment at admission; (Step 2) dynamic monitoring over the initial 72 h (extended to day 7 for VSR/PMR) through three parallel tracks—clinical, imaging, and biomarker surveillance; (Step 3) integrated risk re-classification driven by specific red-flag triggers; and (Step 4) subtype-specific diagnostic pathways. Color coding indicates risk level: green (low), yellow (intermediate), orange (high), red (critical). Ant-STEMI, anterior ST-elevation myocardial infarction; AMI, acute myocardial infarction; BNP, B-type natriuretic peptide; CCU, coronary care unit; CMR, cardiac magnetic resonance; CRP, C-reactive protein; Echo, echocardiography; FWR, free wall rupture; HR, heart rate; LVEF, left ventricular ejection fraction; MCS, mechanical circulatory support; MDT, multidisciplinary team; MR, mitral regurgitation; NLR, neutrophil-to-lymphocyte ratio; NSAIDs, nonsteroidal anti-inflammatory drugs; O₂, oxygen; PCI, percutaneous coronary intervention; PMR, papillary muscle rupture; Q_p_/Q_s_, pulmonary-to-systemic blood flow ratio; RAASi, renin–angiotensin–aldosterone system inhibitors; sST2, soluble suppression of tumorigenicity 2; VSR, ventricular septal rupture; WBC, white blood cell count; q4–6h, every 4–6 hours; q24h, every 24 hours.

## 5 Research gaps and future directions

The foremost limitation in the field is the absence of large-scale, prospective, multicenter registries with standardized CR phenotyping across subtypes (FWR, VSR, PMR), precise time-to-event documentation, and systematic biobanking for biomarker discovery. As Mao et al. demonstrated, existing prediction models are overwhelmingly derived from single-center retrospective cohorts with low events-per-variable ratios, severely limiting generalizability (4). Future models should adhere to recommended sample size criteria (28), employ penalized regression or machine learning approaches, and undergo rigorous external validation across diverse populations.

Prospective evaluation of multi-biomarker panels—combining inflammatory (NLR, CRP trajectory), ECM degradation (MMP-9, HPSE), and myocardial stress markers (sST2, galectin-3)—specifically for CR prediction represents an urgent priority. Key questions remain regarding optimal sampling time points relative to reperfusion, additive prognostic value beyond established clinical predictors, and cost-effectiveness in routine practice.

AI-integrated clinical decision support tools synthesizing continuous ECG monitoring, serial echocardiographic data, and laboratory trajectories into real-time risk scores hold substantial promise, although the extremely low CR event rate (<2%) poses a fundamental challenge for algorithm training that may be addressable through federated learning across centers. Concurrently, early post-reperfusion CMR assessment of IMH and MVO warrants evaluation as a means to define a “rupture-prone phenotype” (11, 25). The optimal frequency and duration of serial echocardiographic surveillance in high-risk patients—a key operational component of our proposed framework—also requires prospective evaluation. From a therapeutic perspective, whether cardioprotective agents such as SGLT2 inhibitors (29) and anti-inflammatory therapies (30), or selective MMP inhibitors, can reduce CR risk remains speculative and warrants dedicated trial design.

Ultimately, translating risk stratification algorithms into electronic health record–embedded, real-time decision support tools represents the overarching goal. This will require collaborative efforts spanning cardiology, cardiac surgery, critical care medicine, and data science—disciplines that must converge to bridge the persistent gap between statistical risk prediction and timely bedside recognition. The framework proposed in this review is intended as a catalyst for such collaboration, offering a structured foundation for future prospective validation.

## 6 Concluding remarks

Cardiac rupture after AMI remains a low-incidence but high-fatality event whose clinical detection continues to depend largely on vigilant bedside observation. This mini review has organized the available evidence on clinical warning signs, point-of-care imaging cues, and circulating biomarker trajectories into a time- and subtype-aware conceptual framework designed to complement existing statistical prediction models. The proposed four-step algorithm—linking baseline risk assessment, dynamic three-track surveillance, red-flag–triggered re-classification, and subtype-specific diagnostic pathways—is hypothesis-generating and intended to support bedside vigilance rather than serve as a validated clinical protocol. Translating this framework into practice will require prospective multicenter studies with standardized CR phenotyping, systematic biomarker validation against CR-specific endpoints, and rigorous evaluation of AI-assisted decision support tools. We hope this work provides a structured foundation for such collaborative efforts and ultimately contributes to narrowing the persistent gap between risk prediction and timely clinical recognition of this devastating complication.
